# Molecular Spectrum of α-Thalassemia Mutations in Antalya, Türkiye and Their Relationship with Hematological Parameters

**DOI:** 10.3390/genes17050543

**Published:** 2026-05-02

**Authors:** Özgür Erkal, Barış Paksoy

**Affiliations:** Department of Medical Genetics, Antalya Training and Research Hospital, Antalya 07100, Türkiye; drbarispaksoy@gmail.com

**Keywords:** α-thalassemia, deletion mutations, mean corpuscular volume (MCV), multiplex ligation-dependent probe amplification (MLPA)

## Abstract

Background: Alpha-thalassemia is one of the most common hereditary hemoglobin disorders worldwide and is caused mainly by deletions in the α-globin gene cluster. Understanding the regional mutation spectrum is important for screening programs and genetic counseling. Methods: This retrospective study included 115 patients evaluated for suspected alpha-thalassemia in Antalya, Türkiye. Molecular analysis was performed using multiplex ligation-dependent probe amplification (MLPA) to detect deletions and duplications in the α-globin gene cluster. Hematological parameters and hemoglobin (Hb) fractions were analyzed and compared among mutation groups. Results: The most frequent mutation detected was the −α^3.7^ deletion followed by the (−α)^20.5^ deletion. Patients with compound heterozygous deletions demonstrated lower Hb, mean corpuscular volume (MCV), and mean corpuscular hemoglobin (MCH) values compared with other groups. Significant correlations were observed between Hb levels and red blood cell (RBC), MCV, and MCH, while red cell distribution width (RDW) showed an inverse relationship. Conclusions: The results demonstrate that −α^3.7^ and (−α)^20.5^ are the predominant α-globin gene variants in the Antalya region. These findings contribute to the characterization of the α-thalassemia mutation spectrum in a clinical cohort and may help improve carrier screening strategies, prenatal diagnosis programs, and genetic counseling services.

## 1. Introduction

Hemoglobin is a molecule responsible for oxygen transport and is composed of heme and globin proteins. In healthy adults, the main hemoglobin types are HbA, HbA_2_, and HbF, all of which share a common alpha chain. The α-globin genes are located on chromosome 16 [1,2,3,4,5,6].

α-thalassemia is an inherited disorder caused by deletional or non-deletional mutations in the α-globin gene cluster, leading to reduced or absent α-globin chain production. Its clinical spectrum ranges from silent carrier states to severe forms such as HbH disease and Hb Bart’s hydrops fetalis [7,8].

The prevalence of α-thalassemia is higher in regions such as Southeast Asia, the Mediterranean, and the Middle East, which is thought to be related to selective advantage against malaria [9].

α-thalassemia results from both deletional and non-deletional mutations. Deletional mutations are the most common, whereas non-deletional variants may be associated with more severe phenotypes and often involve the *HBA2* gene [9]. The most frequent single-gene deletions are −α^3.7^ and −α^4.2^, while common double-gene deletions include (−α)^20.5^, −−SEA,−−MED, −−THAI, and −−FIL [6]. Non-deletional variants such as Hb Constant Spring, Hb Quong Sze, and Hb Adana have been reported, particularly in Southeast Asia [8,10].

Although numerous studies have investigated α-thalassemia, regional differences in mutation distribution and their hematological effects remain important, especially in genetically heterogeneous populations such as Türkiye.

Therefore, this study aims to characterize the distribution of α-thalassemia mutations in Antalya, Türkiye, and to evaluate their associations with hematological parameters and hemoglobin electrophoresis findings. We hypothesize that different α-thalassemia genotypes are associated with distinct hematological profiles that may support clinical interpretation and improve diagnostic approaches.

## 2. Materials and Methods

### 2.1. Patient Screening and Selection

Patients were retrospectively recruited from individuals referred by the hematology clinic for complete blood count abnormalities (low Hb, MCV, and MCH) and/or a family history of α-thalassemia. A total of 115 patients with detected α-globin gene mutations or polymorphisms were included in the study. Only patients with α-globin gene variants detected by MLPA were included, while mutation negative individuals were not evaluated. Due to the retrospective study design, the total number of tested patients could not be reliably determined. Molecular analysis was performed using the MLPA® technique using the SALSA MLPA Probemix P140 HBA kit (MRC Holland, Amsterdam, The Netherlands). Only individuals with identified variants were included, while mutation-negative cases were not evaluated. Clinical and laboratory data were reviewed to exclude common causes of microcytosis and anemia, such as iron deficiency, inflammatory conditions, recent blood transfusion, and pregnancy. MLPA analysis is effective for detecting large deletions and duplications; however, it cannot identify point mutations or small sequence variations.

The presence of beta thalassemia in the patients included in the study was excluded using whole gene sequencing analysis of the *HBB* gene. Hemoglobin concentrations and red blood cell indices were measured with the cobas® 6000 automated analyzer (Roche Diagnostics, Mannheim, Germany). Hemoglobin quantification was performed via automated high-performance liquid chromatography (HPLC).

This retrospective study was conducted in accordance with the Declaration of Helsinki and was approved by the Antalya Training and Research Hospital Clinical Research Ethics Committee on 6 June 2024 (decision no: 8/22). Written informed consent was obtained from all participants prior to their inclusion in the study.

### 2.2. DNA Purification

DNA isolation was performed using 200 µL of peripheral blood collected in an ethylenediaminetetraacetic acid (EDTA) tube. The QIAamp DNA^®^ Micro Kit spin-column method was used. DNA to be used for MLPA analysis was quantified using the QUBIT ONE dsDNA System on a Promega Qubit fluorometer, and the concentration was adjusted to 50 ng.

### 2.3. MLPA Analysis

MLPA analysis was performed using the SALSA MLPA Probemix P140 *HBA* kit (MRC Holland, Amsterdam, The Netherlands), which enables simultaneous detection of common *HBA1* and *HBA2* deletions and duplications across the α-globin gene cluster and the regulatory region (MCS-R2/HS-40). Fragment analysis was carried out according to the manufacturer’s protocol, and results were interpreted using Coffalyser.Net software (version 220513.1739; MRC Holland, Amsterdam, The Netherlands).

### 2.4. Statistical Analysis

Descriptive statistics were expressed as median (minimum–maximum), percentage, and frequency. Differences among patient groups were analyzed using the Kruskal–Wallis test, and post hoc pairwise comparisons were performed with the Bonferroni method. Group differences in categorical variables were assessed with the chi-square test. Relationships among continuous variables were evaluated using Spearman’s rank correlation. A *p*-value < 0.05 was considered statistically significant. All analyses were performed using SPSS version 25.0 (IBM Corp., Armonk, NY, USA).

## 3. Results

A total of 115 patients referred by the hematology clinic with suspected α-thalassemia disease or carrier status, in whom α-globin gene mutations or polymorphisms were detected by MLPA analysis, were included in the study. Only individuals with identified variants were included, and mutation-negative cases were not evaluated.

α-thalassemia variants were evaluated under four categories. Patients were classified into: Group 1, heterozygous or biallelic deletions; Group 2, compound heterozygous deletions; Group 3, duplications; and Group 4, polymorphisms. Patients were grouped based on the type of genetic alteration to allow comparison of hematological parameters across biologically relevant categories. Polymorphic variants were grouped together due to their generally benign or low clinical impact and to allow statistical comparison, given the limited sample size of individual subtypes. These variants are not known to cause α-thalassemia on their own but may represent background genetic variation or be detected in linkage with other alleles. Therefore, although included for comparative purposes, their clinical interpretation differs from pathogenic deletional variants and should be considered with caution.

Of the 115 patients, 57 (49.6%) were male and 58 (50.4%) were female. The mean age was 29.47 ± 15.74 years (range 3–74). Group distribution was as follows: Group 1, n = 84 (73%); Group 2, n = 15 (13%); Group 3, n = 3 (2.6%); and Group 4, n = 13 (11.4%) (Table 1).

In Group 1, various deletion types were identified, including−α^3.7(A)^ heterozygous deletion, −α^3.7(D)^/−α^3.7(D)^ biallelic deletion, −−SEA deletion with Asian polymorphism, −α^3.7(D)^ heterozygous deletion, −α^3.7(F)^ heterozygous deletion, and −α^4.2(C)^ heterozygous deletion. Patients with heterozygous deletions exhibited higher hemoglobin values compared to those with --MED2 deletion, −α^3.7(F)^/ −α^3.7(F)^ biallelic deletion, and −−DUTCH1 deletion mutations (Table 2).

In our study, the most frequently observed mutation in Group 1 was the -α^3.7(D)^ heterozygous deletion, followed by (−α)^20.5^ and −α^3.7(D)^/ −α^3.7(D)^. Other variants were observed at lower frequencies (Table 3)

In Group 2, relatively lower hemoglobin values were observed in patients with the *−α*^3.7(D)^/−−^SEA^ deletion, while relatively higher values were observed in the −*α*^3.7(F)^/(−*α*)^20.5^ mutation (Table 4).

In Group 2, the most common mutation was −α^3.7(D)^/(−α)^20.5,^ followed by −α^3.7(D)^/−−MED1, while other variants were observed at lower frequencies (Table 5)

In Group 3, hemoglobin levels were higher in patients with the ααα anti 3.7(F) duplication mutation compared to those with the ααα anti 3.7(A) duplication mutation (Table 6).

In our study, the most frequently observed mutation in Group 3 was the ααα anti 3.7(F) duplication mutation at 66.7%, followed by the ααα anti 3.7(A) duplication mutation at 33.3% (Table 7).

Hematological parameters varied across subgroups in Group 4 (Table 8). In this group, Asian polymorphism was the most common variant, followed by Polym 4A, while other polymorphisms were observed at lower frequencies (Table 9).

Hb levels were observed to differ significantly among the study groups. Hb levels differed significantly among the study groups (*p* = 0.01), with lower values observed in Groups 1 and 2 compared to Groups 3 and 4. RBC count ×10,000 measurements did not show significant differences among patients in Groups 1–4 (*p* = 0.17, *p* > 0.05). MCV levels were significantly different among the study groups, with lower MCV values in Groups 1 and 2 compared to Groups 3 and 4 (*p* = 0.01). Similarly, MCH levels differed significantly, with Groups 1 and 2 exhibiting lower MCH values than Groups 3 and 4 (*p* = 0.01). RDW levels were significantly different, with higher values observed in Group 2 compared to Groups 1, 3, and 4 (*p* = 0.01). HbA2 levels also differed significantly, with lower values in Group 2 compared to Groups 1, 3, and 4 (*p* = 0.01). HbF and HbA measurements did not differ significantly among the groups (*p* = 0.85 and *p* = 0.71, respectively) (Table 10).

In Group 1, a positive correlation was observed between Hb and RBC, MCV, and MCH values, while a negative correlation was noted with RDW (*p* = 0.01). Negative correlations were observed between HbA2 and MCV, MCH and RDW, and between HbA2 and HbF (*p* = 0.01). No significant correlations were detected with other measurements (*p* > 0.05) (Table 11).

In Group 2, Hb showed a positive correlation with RBC and a negative correlation with RDW (*p* = 0.01). Negative correlations were also observed between HbA2 and RDW, and between MCH and RDW (*p* = 0.01), while MCH and MCV were positively correlated (*p* = 0.01). No other significant correlations were detected (*p* > 0.05) (Table 11).

Due to the very small sample size (n = 3), correlation results in Group 3 should be interpreted with caution (Table 11).

In Group 4, Hb showed a positive correlation with RBC and MCH and a negative correlation with RDW (*p* = 0.01). MCH and MCV were positively correlated, while both showed negative correlations with RDW (*p* = 0.01). A negative correlation was also observed between MCV and RDW (*p* = 0.01). No significant correlations were observed with other measurements (*p* > 0.05) (Table 11).

The most frequent allele was the − α^3.7^ deletion, followed by (−α)^20.5^, while other variants were observed at lower frequencies (Table 12).

## 4. Discussion

Deletional variants constitute approximately 80–90% of α-thalassemia mutations worldwide, particularly in Mediterranean populations, making deletion-focused diagnostic approaches such as MLPA highly effective for regional screening strategies [11].

This study provides a descriptive analysis of α-thalassemia mutations in a cohort of patients from Antalya, focusing on both their distribution and their correlations with hematological parameters and Hb electrophoresis results. By identifying common deletions such as −α^3.7^, (−α)^20.5^, and −−MED1, along with their groups, the study provides descriptive information on the mutation spectrum observed in this cohort. Furthermore, by examining the relationship between genetic findings and hematological indices, the study provides additional descriptive information on genotype–phenotype associations, which may be relevant for genetic counseling and screening considerations. These findings also provide a basis for comparison with other cohort-based molecular studies with other geographical regions.

A study by Divashini Vijian et al., involving 131 individuals in Malaysia suspected of having α-thalassemia based on hematological characteristics, identified the most prevalent deletion mutation as −α^3.7^/αα at 15.4%, followed by −−SEA/αα at 7.4% and −α^4.2^/αα at 3.7% [12]. Similarly, –α^3.7^ was also the predominant mutation in our study, consistent with previous reports. In addition, our study provides a more detailed evaluation by analyzing –α^3.7^ deletion subtypes (D, A, and F), which have not been separately assessed in most previous studies. Furthermore, other common deletion types, including (−α)^20.5^, were also observed, supporting the established mutation spectrum of α-thalassemia.

A study in Malaysia by Divashini Vijian et al. identified −−SEA/−α^3.7^ as the predominant compound heterozygous mutation [12]. In contrast, our findings indicate a different distribution pattern, with −α^3.7^ combined with (−α)^20.5^ or −−MED1 being more frequently observed, suggesting regional variability in compound genotypes. A study by Keser et al. in the Antalya region also reported−α^3.7^ as the predominant genotype, followed by −−MED1 [13]. Consistent with this study, −α^3.7^ remained the most common genotype in our cohort. However, differences in the relative distribution of other genotypes were observed, which may reflect cohort-specific variation.

When investigating allele frequencies in our study, the most common α-globin gene mutation allele was −α^3.7^. Consistent with our study, Öztürk et al. from Istanbul Province; Barış et al. from the Western Aegean; Keser et al. from Antalya; Demir et al. from Thrace, Çelik et al. from Hatay; Güvenç et al. from Adana; Onay et al. from the Aegean; and Karaer et al. from Denizli also reported −α^3.7^ as the most common allele [13,14,15,16,17,18,19,20]. The second most common α-globin gene mutation allele in our study was (−α)^20.5^. Keser et al., Demir et al., Onay et al., Karakaş et al., and Karaer et al. also found (−α)^20.5^ to be the second most common allele [13,18,19,20,21]. The third most common α-globin gene mutation allele in our study was −−MED1. Similarly, Keser et al., Demir et al., Onay et al., Karaer et al., and Karakaş et al. also reported −−MED1 among the most common alleles [13,16,19,20,21]. Öztürk et al., Barış et al., Çelik et al., and Güvenç et al., unlike our study, found −−MED1 to be the second most common α-globin gene mutation allele [15,16,18,19]. In our study, the fourth most common α-globin gene mutation allele was -α^4.2^. Öztürk et al. and Karaer et al. similarly reported−α^4.2^ as the fourth most common allele [15,21]. The fifth most common mutation allele in our study was ααα anti 3.7, followed by −−DUTCH1, −−MED2, and −−SEA. A study from Thrace by Demir et al. also reported ααα anti 3.7 among the more frequent mutation alleles [16].

This study provides a detailed evaluation of the hematological effects of −α3.7 deletion subtypes (D, F, and A), which have been less frequently analyzed separately in previous studies. Our findings indicate that certain subtypes, particularly biallelic forms, may be associated with more pronounced reductions in Hb levels compared to others. Higher Hb values were generally observed in heterozygous deletion mutations compared to biallelic deletions. There is a relationship between α-thalassemia mutations and hematological parameters. A decrease in the number of alpha-globin genes is associated with lower MCV and MCH values. Similarly, Güvenç et al., Karaer et al., El-Kalla, and Baysal et al. [18,20,22] reported that a reduction in the number of alpha-globin genes was correlated with decreased MCV and MCH values, consistent with our findings. Overall, these findings support the relationship between gene dosage and hematological severity in α-thalassemia.

This study is strong in terms of comprehensively evaluating the distribution of α-thalassemia mutations in the Antalya region and their relationship with hematological parameters. The inclusion of a large patient cohort and detailed molecular analyses of various deletion subtypes, such as −α^3.7^, (−α)^20.5^, and −−MED1, allows for a more accurate characterization of the mutation spectrum within this cohort. Furthermore, the comparison of identified mutations with both hematological indices and Hb electrophoresis results provides information that may be relevant for clinical practice and genetic counseling. From a clinical perspective, our findings provide practical value in the evaluation of microcytic anemia. The associations between α-thalassemia genotypes and hematological parameters (Hb, MCV, MCH, and RDW) may support differential diagnosis, particularly in settings where molecular testing is limited. Additionally, the more pronounced hematological alterations observed in compound heterozygous mutations may help prioritize patients for advanced molecular testing. This approach may contribute to optimizing carrier screening and prenatal diagnostic strategies. By integrating mutation subtypes with hematological data, this study provides descriptive information on genotype–phenotype associations beyond mutation frequency reporting. These findings support a genotype–phenotype relationship rather than a purely descriptive mutation profile.

However, the study has several limitations. First, the sample was collected exclusively from the Antalya region, limiting its generalizability to the entire country. Additionally, the sample size may have been insufficient to detect rare mutations, potentially underestimating their actual prevalence. In addition, potential confounding factors affecting hematological parameters, such as iron deficiency or other comorbid conditions, could not be fully controlled due to the retrospective design. This may have influenced the interpretation of genotype–phenotype correlations. The small sample size in certain subgroups (e.g., Group 3) may limit statistical power and increase the risk of type I and type II errors; therefore, subgroup analyses should be interpreted with caution. Due to the lack of HBA gene sequencing analysis, rarer mutations, such as small deletions, insertions, or point mutations, could not be detected. The absence of a healthy control group limits the ability to assess the absolute impact of the detected mutations and may affect the interpretation of differences between groups. Furthermore, as only MLPA-positive patients were included, the findings reflect the distribution of variants in a selected cohort and cannot be interpreted as population prevalence. The retrospective nature of data collection and the restriction of genetic analyses to a specific platform or methodology may limit the overall applicability of the results. These findings should be interpreted as exploratory due to small sample sizes, particularly for very small subgroups. These strengths and limitations should be considered when interpreting the study findings clinically and epidemiologically.

From a clinical perspective, defining the regional mutation spectrum of alpha-thalassemia is particularly important for optimizing premarital screening and prenatal diagnostic programs in Mediterranean populations. In regions such as Antalya where migration and genetic heterogeneity are increasing, molecular diagnostic approaches such as MLPA can significantly improve diagnostic accuracy and enable earlier identification of at-risk couples. Future multicenter studies including larger cohorts and full *HBA1 and HBA2* gene sequencing may further clarify the contribution of rare non-deletional variants to the regional disease burden.

From a practical perspective, our findings may support clinical decision-making in patients with microcytic anemia. In cases with low MCV and MCH but normal iron status, α-thalassemia should be considered, particularly in regions with a high mutation prevalence, such as the Mediterranean region. Patients showing more pronounced hematological alterations may benefit from prioritized molecular testing. These findings may help guide more efficient use of genetic testing and improve early identification of carriers.

Previous studies in Türkiye and Mediterranean populations have shown that most α-thalassemia cases are caused by large deletional variants such as −α^3.7^, −α^4.2^ and (−α)^20.5^, which can be reliably detected using MLPA analysis. During the study period, sequencing facilities were not available in our laboratory; therefore, the molecular investigation focused primarily on deletional variants that represent the most frequent causes of α-thalassemia in the regional population.

## 5. Conclusions

This study provides a descriptive analysis of α-thalassemia variants in a cohort of patients from Antalya. The most frequently observed variants were consistent with those previously reported in the literature. The identification of subtypes of the −α^3.7^ deletion variant (−α^3.7(D)^, −α^3.7(A)^, and −α^3.7(F)^) allowed for a more detailed description of the mutation spectrum within this cohort. In addition, other variants such as (−α)^20.5^, −−MED1, −α^4.2^, and ααα anti 3.7 were also observed.

These findings are consistent with existing data and provide additional descriptive information on MLPA-detected variants and their hematological features in a selected clinical cohort. The results should be interpreted cautiously and do not imply population-level prevalence or mechanistic conclusions.

## Figures and Tables

**Table 1:** Demographic characteristics of the study groups (median [min–max]).

Group	Male n (%)	Female n (%)	Age (Years)
Group 1 (heterozygous/biallelic deletions)	43 (75.4)	41 (70.7)	28 (3–66)
Group 2 (compound heterozygous deletions)	5 (8.8)	10 (17.2)	36 (6–74)
Group 3 (duplications)	2 (3.5)	1 (1.7)	38 (33–49)
Group 4 (polymorphisms)	7 (12.3)	6 (10.3)	29 (8–42)

**Table 2:** Hematological parameters in Group 1 (median [min–max]).

Genotype	Hb (g/dL)	RBC (×10^6^/µL)	MCV (fL)	MCH (pg)	RDW-CV (%)	HbA2 (%)	HbF (%)	HbA (%)
−−DUTCH1	11.55 (11.0–12.1)	5.25 (4.3–6.2)	71.9 (65.6–78.2)	22.55 (19.6–25.5)	16.45 (14.3–18.6)	0.03 (0.03–0.03)	0.01 (0.01–0.01)	0.87 (0.87–0.87)
−−MED1	11.7 (11.1–14.0)	5.65 (5.3–6.9)	68.6 (58.3–70.0)	21.05 (19.1–23.0)	15.7 (14.0–19.0)	0.03 (0.02–0.03)	0.01 (0–0.01)	0.86 (0.83–0.88)
−*α*^3.7(A)^ heterozygous deletion	13.0 (12.2–14.6)	5.2 (5.1–5.61)	78.1 (68.1–78.4)	24.1 (22.3–33.3)	15.4 (14.0–16.9)	0.03 (0.03–0.03)	0.00 (0–0.01)	0.86 (0.86–0.87)
−*α*^3.7(D)^/−*α*^3.7(D)^ biallelic deletion	12.2 (10.4–14.1)	5.6 (4.8–6.2)	67.5 (62.7–82.4)	20.7 (19.8–26.1)	15.5 (13.9–18.3)	0.03 (0.02–0.03)	0.01 (0–0.04)	0.87 (0.78–0.88)
(−*α*)^20.5^ heterozygous deletion	11.1 (9.2–13.6)	5.4 (4.5–6.9)	64.2 (59.1–71.0)	20.1 (17.2–30.2)	16.3 (14.0–27.5)	0.03 (0.01–0.03)	0.01 (0–0.03)	0.86 (0.77–0.97)
−−MED2	11.3 (11.3–11.3)	5.1 (5.1–5.1)	69.8 (69.8–69.8)	22.2 (22.2–22.2)	17.2 (17.2–17.2)	0.03 (0.03–0.03)	0.02 (0.02–0.02)	0.84 (0.84–0.84)
−−^SEA^ + Asian polymorphism	13.8 (13.8–13.8)	5.18 (5.18–5.18)	85.5 (85.5–85.5)	26.6 (26.6–26.6)	13.1 (13.1–13.1)	0.03 (0.03–0.03)	0.01 (0.01–0.01)	0.86 (0.86–0.86)
*−α*^3.7(D)^ heterozygous deletion	13.0 (10.0–15.2)	5.4 (4.4–6.6)	70.9 (63.7–87.3)	23.4 (19.3–30.3)	15.6 (13.1–18.8)	0.03 (0.01–0.04)	0.01 (0–0.03)	0.86 (0.82–0.88)
*−α*^3.7(F)^/−*α*^3.7(F)^ biallelic deletion	10.7 (8.8–12.6)	5.45 (4.8–6.1)	64.4 (62.6–66.1)	19.45 (18.3–20.6)	16.35 (14.7–18.0)	0.03 (0.03–0.03)	0.005 (0–0.01)	0.875 (0.87–0.88)
*−α*^3.7(F)^ heterozygous deletion	13.05 (12.9–13.2)	5.45 (5.0–5.9)	76.3 (68.7–83.9)	24.1 (21.9–26.3)	13.9 (13.6–14.2)	0.02 (0.02–0.02)	0.01 (0.01–0.01)	0.845 (0.82–0.87)
*−α*^4.2(C)^ heterozygous deletion	13.1 (11.9–14.1)	5.0 (4.8–5.3)	80.35 (77.1–84.1)	25.7 (24.8–27.6)	13.5 (13.0–15.5)	0.025 (0.02–0.03)	0.01 (0.01–0.05)	0.81 (0.76–0.87)

**Table 3:** Distribution of mutations in Group 1.

Genotype	n	%
*−α* ^3.7(D*)*^ */αα*	29	34.5
*(−α)*^20.5^/*αα*	23	27.4
−*α*^3.7(D)^/−*α*^3.7(D)^	9	10.7
−−^MED1^	8	9.5
*−α* ^4.2^ */αα*	4	4.8
*−α* ^3.7*(A)*^ */αα*	3	3.6
*−α* ^3.7*(F)*^ */αα*	2	2.4
−−^DUTCH1^	2	2.4
−−^MED2^	1	1.2
−−^SEA^ + Asian polymorphism	1	1.2
−*α*^3.7(D)^/−*α*^3.7(F)^	1	1.2
−*α*^3.7(F)^/−*α*^3.7(F)^	1	1.2

**Table 4:** Hematological parameters in Group 2 (median [min–max]).

Genotype	Hb (g/dL)	RBC (×10^6^/µL)	MCV (fL)	MCH (pg)	RDW-CV (%)	HbA2 (%)	HbF (%)	HbA (%)
−*α*^3.7(F)^/(−*α*)^20.5^	9.1 (9.1–9.1)	5.2 (5.2–5.2)	56.4 (56.4–56.4)	17.6 (17.6–17.6)	23.1 (23.1–23.1)	0.02 (0.02–0.02)	0.05 (0.05–0.05)	0.83 (0.83–0.83)
*(−α)*^20.5^/−*α*^4.2(C)^	8.5 (8.5–8.5)	5.5 (5.5–5.5)	52.5 (52.5–52.5)	15.3 (15.3–15.3)	26.1 (26.1–26.1)	0.02 (0.02–0.02)	0.01 (0.01–0.01)	0.89 (0.89–0.89)
*−α*^3.7(D)^/−−^SEA^	7.7 (7.7–7.7)	4.1 (4.1–4.1)	64.3 (64.3–64.3)	18.6 (18.6–18.6)	28.8 (28.8–28.8)	0.02 (0.02–0.02)	0.00 (0–0)	0.91 (0.91–0.91)
−*α*^3.7(B)^/−−^MED2^	7.9 (7.9–7.9)	4.2 (4.2–4.2)	65.2 (65.2–65.2)	18.9 (18.9–18.9)	28.9 (28.9–28.9)	0.01 (0.01–0.01)	0.00 (0–0)	0.92 (0.92–0.92)
*−α*^3.7(D)^/−−^MED1^	10.5 (9.0–11.8)	5.5 (4.5–6.9)	58.1 (56.3–63.0)	18.2 (17.1–20.3)	26.9 (21.2–31.2)	0.02 (0.01–0.04)	0.01 (0–0.01)	0.89 (0.82–0.90)
−*α*^3.7(D)^/*(−α)*^20.5^	8.95 (7.7–10.0)	5.0 (4.2–5.9)	59.4 (52.5–69.3)	18.1 (16.9–19.1)	27.6 (21.8–30.5)	0.02 (0.01–0.05)	0.01 (0–0.02)	0.845 (0.82–0.92)

**Table 5:** Distribution of mutations in Group 2.

Genotype	n	%
−α^3.7(D)^/(−α)^20.5^	6	40.0
−α^3.7(D)^/ −−^MED1^	5	33.3
−α^3.7(B)^/ −−^MED2^	1	6.7
−α^3.7(F)^/(−α)^20.5^	1	6.7
−α^3.7(D)^/−−^SEA^	1	6.7
(−α)^20.5^/−α^4.2(C)^	1	6.7

**Table 6:** Hematological parameters in Group 3 (median [min–max]).

Genotype	Hb (g/dL)	RBC (×10^6^/µL)	MCV (fL)	MCH (pg)	RDW-CV (%)	HbA2 (%)	HbF (%)	HbA (%)
ααα anti 3.7(F)	13.65 (12.4–14.9)	5.46 (5.0–5.92)	74.15 (65.9–82.4)	25.3 (20.9–29.7)	14.55 (11.9–17.2)	0.035 (0.03–0.04)	0.01 (0.01–0.01)	0.86 (0.85–0.87)
ααα anti 3.7(A)	11.9 (11.9–11.9)	4.5 (4.5–4.5)	82.0 (82.0–82.0)	26.7 (26.7–26.7)	13.8 (13.8–13.8)	0.03 (0.03–0.03)	0.01 (0.01–0.01)	0.86 (0.86–0.86)

Hb: Hemoglobin, Hct: Hematocrit, MCV: Mean Corpuscular Volume, MCH: Mean Corpuscular Hemoglobin, MCHC: Mean Corpuscular Hemoglobin concentration, RDW: Red cell distribution width, RBC: Red blood cell.

**Table 7:** Distribution of mutations in Group 3.

Genotype	n	%
ααα anti 3.7 (F)	2	66.7%
ααα anti 3.7(A)	1	33.3%

**Table 8:** Hematological parameters in Group 4 (median [min–max]).

Genotype	Hb (g/dL)	RBC (×10^6^/µL)	MCV (fL)	MCH (pg)	RDW-CV (%)	HbA2 (%)	HbF (%)	HbA (%)
Asian polymorphism + Polym 3B	14.95 (13.5–16.4)	5.31 (4.52–6.1)	81.1 (76.5–85.6)	28.4 (26.9–29.9)	12.3 (11.5–13.1)	0.03 (0.02–0.03)	0.01 (0.01–0.01)	0.86 (0.84–0.88)
Asian polymorphism	14.3 (13.8–15.8)	5.3 (4.8–5.8)	81.4 (76.2–90.6)	27.2 (25.6–32.2)	12.7 (11.9–13.2)	0.03 (0.02–0.03)	0.01 (0–0.01)	0.86 (0.83–0.88)
Polym 3A	17.2 (17.2–17.2)	5.7 (5.7–5.7)	81.4 (81.4–81.4)	30.2 (30.2–30.2)	11.7 (11.7–11.7)	0.02 (0.02–0.02)	0.01 (0.01–0.01)	0.86 (0.86–0.86)
Polym 3A + Asian polymorphism + Polym 3B	15.3 (15.3–15.3)	5.4 (5.4–5.4)	83.7 (83.7–83.7)	28.3 (28.3–28.3)	13.0 (13.0–13.0)	0.01 (0.01–0.01)	0.00 (0–0)	0.83 (0.83–0.83)
Polym 4A	12.65 (8.4–14.3)	4.8 (4.3–5.08)	80.9 (67.8–87.7)	27.4 (17.5–28.9)	13.3 (13.1–19.8)	0.03 (0.01–0.03)	0.01 (0–0.01)	0.88 (0.77–0.93)

**Table 9:** Distribution of variants in Group 4.

Genotype	n	%
Asian polymorphism	5	38.5
Polym 4A	4	30.8
Asian polymorphism + Polym 3B	2	15.4
Polym 3A	1	7.7
Polym 3A + Asian polymorphism + Polym 3B	1	7.7

**Table 10:** Comparison of hematological parameters across groups (median [min–max]).

Parameter	Group 1 (n = 84)	Group 2 (n = 15)	Group 3 (n = 3)	Group 4 (n = 13)	*p*-Value **
Hb (g/dL)	11.9 (8.8–15.2)	9.1 (7.7–11.8)	12.4 (11.9–14.9)	14.3 (8.4–17.2)	0.01 *
RBC (×10^6^/µL)	5.40 (4.3–6.9)	5.20 (4.10–6.90)	5.00 (4.50–5.92)	5.10 (4.30–6.10)	0.17
MCV (fL)	68.75 (58.3–87.3)	58.1 (52.5–69.3)	82.0 (65.9–82.4)	81.4 (67.8–90.6)	0.01 *
MCH (pg)	21.65 (17.2–33.3)	18.2 (15.3–20.3)	26.7 (20.9–29.7)	28.2 (17.5–32.2)	0.01 *
RDW-CV (%)	15.7 (13.0–27.5)	27.3 (21.2–31.2)	13.8 (11.9–17.2)	13.1 (11.5–19.8)	0.01 *
HbA2 (%)	0.03 (0.01–0.04)	0.02 (0.01–0.05)	0.03 (0.03–0.04)	0.03 (0.01–0.03)	0.01 *
HbF (%)	0.01 (0–0.05)	0.01 (0–0.05)	0.01 (0.01–0.01)	0.01 (0–0.01)	0.85
HbA (%)	0.86 (0.76–0.97)	0.89 (0.82–0.92)	0.86 (0.85–0.87)	0.86 (0.77–0.93)	0.71

* Statistically significant values (*p* < 0.05) ** Kruskal–Wallis test, µ = median value, min = minimum value, Max = maximum value.

**Table 11:** Examination of the Relationships Between Measurements.

Groups	r	Hb gr/dL	RBC (×10^6/mm^3^)	MCV um^3^	MCH pg	RDW-CV	HbA2%	HbF %	HbA %
Group 1	Hb g/dL	1							
	RBC (×10^6^/µL)	0.661 **	1						
	MCV fL	0.621 **	−0.304 **	1					
	MCH pg	0.643 **	−0.204	0.823 **	1				
	RDW-CV	−0.521 **	0.022	−0.472 **	−0.484 **	1			
	HbA2%	0.105	0.036	0.093	0.059	−0.336 **	1		
	HbF%	0.050	−0.073	0.164	0.045	−0.014	−0.034	1	
	HbA%	−0.102	0.107	−0.130	−0.002	0.128	0.215	−0.463 **	1
Group 2	Hb g/dL	1							
	RBC (×10^6^/µL)	0.903 **	1						
	MCV fL	−0.196	−0.461	1					
	MCH pg	−0.214	−0.604 *	0.664 **	1				
	RDW-CV	−0.379	−0.339	0.065	0.024	1			
	HbA2	0.206	0.239	−0.245	−0.046	−0.657 **	1		
	HbF	0.037	0.078	−0.221	−0.098	−0.466	0.206	1	
	HbA	−0.116	−0.041	0.373	−0.079	0.301	−0.071	−0.473	1
Group 3	Hb g/dL	1							
	RBC (×10^6^/µL)	−0.013	1						
	MCV fL	0.379	−0.930	1					
	MCH pg	0.651	−0.767	0.949	1				
	RDW-CV	−0.666	0.754	−0.943	0.949	1			
	HbA2	0.988	−0.168	0.518	0.761	−0.774	1		
	HbF	0.01	0.01	0.01	0.01	0.01	0.01		
	HbA	−0.778	0.639	−0.876	−0.984	0.987	−0.866	0.01	1
Group 4	Hb g/dL	1							
	RBC (×10^6^/µL)	0.668 *	1						
	MCV fL	0.539	−0.212	1					
	MCH pg	0.760 **	0.026	0.913 **	1				
	RDW-CV	−0.808 **	−0.206	−0.756 **	−0.908 **	1			
	HbA2%	−0.237	0.056	−0.385	−0.356	0.192	1		
	HbF%	−0.215	−0.178	−0.313	−0.127	0.134	0.286	1	
	HbA%	0.119	0.039	0.264	0.123	0.008	−0.202	−0.358	1

Spearman correlation test. ** significant relationship at the 0.01 level, * significant relationship at the 0.05 level, r = correlation coefficient ((−)1.00–1.00).

**Table 12:** Distribution of α-thalassemia variant allele frequencies in the study cohort (%).

HBA Variants	Distribution of α-Thalassemia Variant Allele Frequencies in the Study Cohort (%)
−*α*^3.7^	29.5
(−*α*)^20.5^	13
−−^MED1^	5.65
−*α*^4.2^	2.1
ααα anti 3.7	1.3
−−^DUTCH1^	0.87
−−^MED2^	0.87
−−^SEA^	0.87
Asian polym	3.9
Polym 4A	1.74
Polym 3B	1.3
Polym 3A	0.87

## Data Availability

The article encompasses the original contributions outlined in the study. Additional enquiries may be sent to the corresponding author.
